# The Association between Cannabis Use, Mental Illness, and Suicidal Behavior: What is the Role of Hopelessness?

**DOI:** 10.3389/fpsyt.2013.00125

**Published:** 2013-10-11

**Authors:** Gianluca Serafini, Maurizio Pompili, Marco Innamorati, Elizabeth C. Temple, Mario Amore, Stefan Borgwardt, Paolo Girardi

**Affiliations:** ^1^Department of Neurosciences, Mental Health, and Sensory Organs, Sant'Andrea Hospital, Sapienza University of Rome, Rome, Italy; ^2^School of Health Sciences, University of Ballarat, Ballarat, VIC, Australia; ^3^Department of Neuroscience, Rehabilitation, Ophthalmology, Genetics, Maternal and Child Health, Section of Psychiatry, University of Genova, Genova, Italy; ^4^Department of Psychiatry, University of Basel, Basel, Switzerland

**Keywords:** cannabis use, major affective disorders, suicidal behavior, hopelessness, adolescence

## Introduction: The Complexity of Cannabis Misuse

Cannabis is one of the most common illegal psychoactive substance used in European countries, in particular among adolescents and young adults (1). It has been estimated that almost 55% of adolescents aged 15–19 years have used cannabis at least once in their lifetime (2), while past year use is reported by approximately 30% of 15–17 year olds and over 47% of those aged 18–19 years (3).

Cannabis use has been associated with several adverse life outcomes including unemployment, legal problems, dependence, early school leaving, increased risk of developing both psychotic and affective disorders (3, 4) together with brain structural and functional abnormalities (5, 6). An association between cannabis use, psychiatric disorders and suicidal behavior has also frequently been reported, although the exact nature of this link is still poorly understood (4).

Globally, suicide is one of the most common causes of death among young people aged 10–24 years (6% of deaths), exceeded only by motor vehicle accidents (10%) (7). Over the last decade suicidal behavior has increased among adolescents and young adults, there has also been a trend toward the earlier initiation of cannabis use (8). This has led researchers to investigate the associations between the two factors to determine if cannabis use may be considered a factor that can trigger suicidal behavior.

Evidence indicates that cannabis use is significantly associated with both attempted and completed suicides among healthy youths (9) and both twin studies (10) and case-control comparisons (11) have shown the increased risk of suicide ideation/attempts in those who use cannabis. Moreover, a longitudinal study found that frequent cannabis use (at least several times a week) predicted later suicidal ideation in susceptible males but not females (12). The earlier that this intense use first occurred and the higher the frequency of cannabis use, faster the susceptible individuals experienced suicidal thoughts.

Frequent and early cannabis use has also been associated with impaired mental wellbeing among young individuals (13, 14), and the risk of developing psychiatric conditions such as psychosis (15) and major affective disorders (16). Specifically, evidence suggests that cannabis use may exacerbate pre-existing conditions such as bipolar disorder, and predict negative outcomes and psychosocial impairment (17, 18). According to longitudinal studies, the high and frequent use of cannabis is also associated with longer recovery times for affective conditions, more hospitalizations, poorer compliance with treatment, increased aggression, and poorer response to treatment in patients with bipolar disorder type I and II (12, 17).

Nevertheless, it is important to note that many of the studies investigating associations between cannabis use and psychiatric conditions are cross-sectional in nature and cannot establish a causal relationship between the two phenomena (19). Further, several studies (20, 21) suggest a bidirectional relationship, as cannabis use variables do not solely explain the psychiatric outcomes observed nor do pre-existing psychiatric conditions fully explain the increased use of cannabis. Some researchers (22) have suggested that individuals with high levels of anxiety sensitivity or hopelessness may be more sensitive to the negative reinforcement processes of substance use (i.e., the ability of substances to modulate negative affective states) than non-affected individuals; however, some individuals experiencing the onset of mania or depression are not more likely to report increased cannabis use than those not experiencing these disorders (23, 24). In addition, other authors (25) have questioned the hypothesis that individuals may use cannabis to self-medicate psychotic or depressive symptoms.

In summary, cannabis use may be considered only as a risk factor, and possibly one of a great many that may predict the onset or exacerbation of affective disorders and suicidal behavior (26). Thus, whether cannabis use can trigger psychiatric disorders or only precipitate or exacerbate psychiatric conditions in vulnerable individuals, is still poorly understood.

## Affective Symptoms and Hopelessness: A Possible Mediating Factor?

Depression, and in particular hopelessness, are widely recognized as strong predictors of suicidal behavior (15, 27–29). Specifically, hopelessness has been shown to predict completed suicides among psychiatric patients after 10–20 years of follow-up (30, 31), and it is significantly associated with both adolescent self-harm and completed suicides (32).

Studies have also reported that hopelessness may be a risk factor of substance use suggesting that the presence of hopelessness could be considered a predictor of substance misuse (33, 34). With regard to cannabis use, Malmberg et al. (22) found that adolescents with high levels of hopelessness were more likely to have ever smoked cannabis when compared to adolescents with lower levels. The authors also suggested that increased levels of hopelessness were usually associated with earlier initiation of cannabis use. As such, it is possible that young adolescents experiencing hopelessness are more likely to use cannabis as a strategy to cope with their negative thoughts and feelings (35).

**Figure 1:**
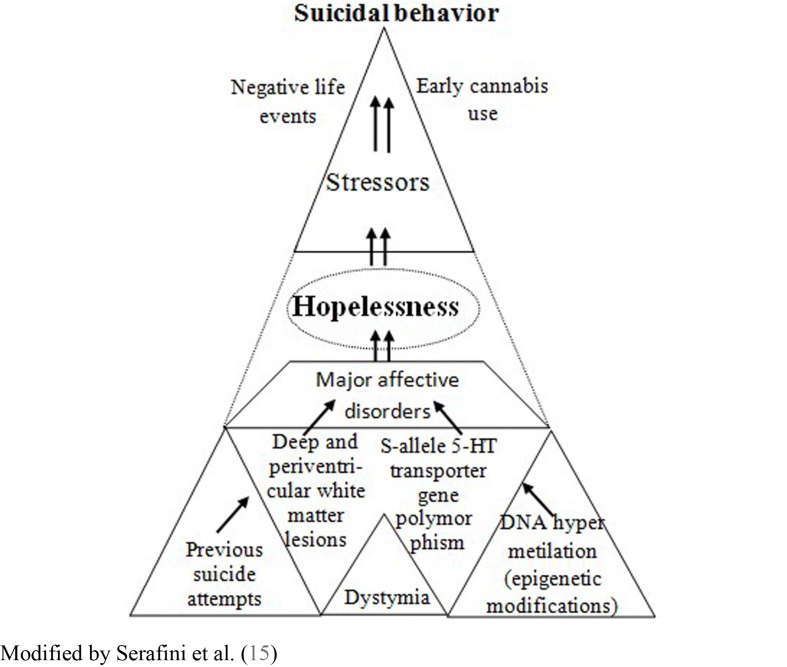
**The complex interaction between risk factors involved in the emergence of suicidal behavior: the mediating effect of hopelessness**.

Informed by such research evidence, we suggest that the presence of hopelessness should be considered as a specific risk factor of negative outcome and suicidal behavior among depressed individuals with a history of early cannabis use. Thus in this review, we propose a theoretical model that addresses this issue (see Figure 1 for more details). This view is consistent with the hypothesis that early cannabis use may represent a relevant risk factor that can trigger or exacerbate suicidal behavior in vulnerable adolescents and young adults, with high hopelessness levels. In addition, vulnerable individuals may show hopelessness (36) and risk factors such as dysthymic temperamental traits (37, 38), dysthymia associated with periventricular white matter abnormalities (39), possibly the S-allele of the serotonin transporter gene polymorphism (5-HTTLPR) (40), sleep disturbances (e.g., insomnia) (41), abnormal pro-inflammatory cytokines levels (42), and/or comorbid symptom development (43). We highly recommend that the complex interaction between these variables is more closely investigated in adolescents at risk, in order to understand the possible emergence of depression and suicide.

However, studies including those informing the development of this model, should be considered in the light of significant shortcomings. Many of the studies were conducted using cross-sectional designs or included retrospective evaluations of lifetime behavior while attempting to predict long-term outcome variables or making reliable causal inferences. In addition, these studies adopted different measurements and outcome variables or they assessed patients at different time points (for more details see a complete list of limitations within Table 1 in Serafini et al. (15)). Further, not all studies included specific follow-up periods and only some of them were able to distinguish between suicide attempts and completions. Furthermore, the use of heterogeneous samples did not permit some researchers to determine a clear association between the onset of psychiatric conditions, suicidal behavior and the age of first cannabis use. Regarding retrospective studies, the absence of any strategies to ensure both inter-rater reliability and validity of the data also indicates that careful consideration must be given to the study results. Finally, the patients did not receive psychiatric assessments using structured psychometric instruments in all studies.

**Table 1:** **Risk factors for suicide risk and early cannabis use in adolescents**.

Socio-demographic and social factors	Death/loss of a parent or close friend
	Social events including humiliation, loss, defeat, or threat
	Interpersonal problems such as romantic difficulties
	Poor social support
	Financial or employment problems
	Availability of weapons
	Occasional failure at school or in society
Parental and family factors	Family history of suicide or suicide attempts
	Family history of violence and aggression
	Parental substance abuse and/or antisocial behavior
	Parental separation or divorce
	An argument with a parent
	Disorganized family environment
	History of physical/sexual abuse as a child or childhood maltreatment
Individual factors	Psychiatric disorders such as affective disorders and psychosis
	Sleep disturbances such as insomnia
	Antisocial and conduct problems
	Loneliness
	Impulsivity and poor self-control
	Hopelessness
	Neuroticism
	Victimization
	History of suicide attempts
	Impairments in decisional competence and decision-making skills
	Aggressive threats/fantasies
	Dysthymic temperamental traits

*Sources: van Ours et al. (12), Malmberg et al. (22), Beautrais et al. (49), Bridge et al. (50), Berger et al. (44), and Reinherz et al. (51)*.

## Implications for Prevention

Psychological distress and social decline need to be carefully investigated in young adolescents in order to provide appropriate ongoing management (44). Youth suicide prevention programs aimed at identifying risk behavior and the subgroups of individuals at high suicidal risk are absolutely necessary in clinical practice. Based on the current literature, such vulnerable subgroups of individuals include those who used cannabis early during adolescence (22), those who currently experience hopelessness (15), and those at high clinical risk of psychiatric conditions (45–47). Furthermore, vulnerable individuals usually present with additional risk factors that may severely influence their childhood development [e.g., a poor performance on tasks assessing sustained attention, impulse control and executive functioning (48)], presumably affecting both their suicide risk as well as early use of cannabis (12, 22, 44, 49–51) (for more details see Table 1).

Early warning signs of emerging psychiatric conditions such as behavioral, emotional, and cognitive changes, should be quickly recognized by clinicians by performing a multi-dimensional assessment of the patients (52). In addition, we recommend the careful assessment of hopelessness since it has been demonstrated to significantly increase the accuracy of suicide risk assessment by allowing the collection of reliable information about suicide risk even several years after the initial assessment (53). We also suggest that clinicians assess the current and past use of cannabis in their patients, including a determination of the age of initial use.

According to the affective model of prevention, young adolescents begin to use cannabis because they have poor self-esteem, poor self-control, and poor decision-making skills (35). In this context, youths may also experience negative expectations about their self and their future related to depression or pervasive feelings of loneliness (54). Prevention programs aimed at helping young adolescents to clarify their subjective states, improve their decision-making abilities and enhance their self-esteem are available, thus potentially preventing the onset of hopelessness and subsequent suicidal ideation (55, 56). Young adolescents are expected to perceive the information provided in these programs as credible, otherwise they will not be likely to modify their behaviors (57). These prevention programs should be conducted during early adolescence and specifically focused on addressing hopelessness, although it is currently unclear whether the benefits may vary for different subgroups of adolescents (e.g., younger or older individuals) (57).

Evidence also suggests that school-based programs are very effective in preventing and/or reducing the use of cannabis among young adolescents, especially if they are able to provide active motivational strategies that inform adolescents about the prejudices against using psychoactive medications (55–57). For example, typical strategies may include actively explaining how to implement non-use behavior, such as coping skills for prodrug pressures and negative affective states, helping youths to understand that most people do not use cannabis, as well as increasing their awareness of the consequences of cannabis use and benefits related to non-use (57). In particular, research has demonstrated the efficacy of social-influence programs that use interactive (not didactic) sessions, and those that encourage active participation in small groups (55, 56).

In summary, clinicians need to be aware of the importance of preventive programs that are directed at preventing/treating modifiable factors such as adolescent hopelessness and/or delaying early cannabis use in specific subgroups of adolescents who experience major affective disorders.

## Conclusion

Suicide, cannabis use, and psychiatric conditions (e.g., depression) are likely to be underpinned by similar complex factors. Of particular interest for clinicians is the identification of individuals at risk of suicide who show early (i.e., prodromal) affective symptoms such as hopelessness. Suicide prevention programs may provide additional benefits if they focus on delaying or reducing adolescent cannabis use as well as responding to early signs of depression and hopelessness, which are widely recognized as important risk factors for suicide (58).
